# Intravascular Ultrasound versus Angiography Guided Drug Eluting Stent Implantation in Patients with Left Main Coronary Artery Disease – A Systematic Review and Meta-Analysis

**DOI:** 10.31083/j.rcm2501032

**Published:** 2024-01-22

**Authors:** Kevin Karim, Mohammad Rizki Akbar, Miftah Pramudyo, Januar Wibawa Martha

**Affiliations:** ^1^Department of Cardiology and Vascular Medicine, Faculty of Medicine, University of Padjadjaran, 40161 Bandung, Indonesia

**Keywords:** angiography, intravascular ultrasound, percutaneous coronary intervention, left main coronary artery disease, mortality, infarction

## Abstract

**Background:**

Several technical limitations exist in angiography procedures, including suboptimal visualization of a particular location and angiography only providing information about the contour of the vascular lumen, while intravascular ultrasound (IVUS) provides information regarding wall composition on coronary vascular lesions. With recent trials demonstrating IVUS benefits over standard angiography, our meta-analysis aimedto evaluate and summarize the current evidence on whether IVUS-guided drug-eluting stent (DES) placement resulted in better outcomes than the angiography-guided DES placement in patients with left main coronary artery (LMCA) disease. This meta-analysis aimed to analyze the current evidence on the IVUS-guided and angiography-guided drug-eluting stent (DES) placement in patients with LMCA disease.

**Methods:**

Literature searching was performed using Scopus, Embase, PubMed, EuropePMC, and Clinicaltrials.gov using PRISMA guidelines. The intervention group in our study are patients undergoing IVUS-guided percutaneous coronary intervention (PCI) and the control group are patients undergoing angiography alone-guided PCI. Cardiovascular mortality, all-cause mortality, target lesion revascularization, myocardial infarction, and stent thrombosis were compared between the two groups.

**Results:**

There were 11 studies comprising 24,103 patients included in this meta-analysis. IVUS-guided PCI was associated with lower cardiovascular mortality (hazard ratio (HR) 0.39 [95% CI 0.26, 0.58], *p* < 0.001; $I^{2}$: 75%, *p* < 0.001) and all-cause mortality (HR 0.59 [95% CI 0.53, 0.66], *p* < 0.001; $I^{2}$: 0%, *p* = 0.45) compared to angiography alone guided PCI. The group receiving IVUS guided PCI has a lower incidence of myocardial infarction (HR 0.66 [95% CI 0.48, 0.90], *p* = 0.008; $I^{2}$: 0%, *p* = 0.98), target lesion revascularization (HR 0.45 [95% CI 0.38, 0.54], *p* < 0.001; $I^{2}$: 41%, *p* = 0.10) and stent thrombosis (HR 0.38 [95% CI 0.26, 0.57], *p* < 0.001; $I^{2}$: 0%, *p* = 0.50) compared to the control group.

**Conclusions:**

Our meta-analysis demonstrated that IVUS-guided DES placement had lower cardiovascular mortality, all-cause mortality, target lesion revascularization, myocardial infarction, and stent thrombosis than angiography-guided DES implantation.

## 1. Introduction

Coronary angiography with the use of contrast media is utilized in cardiac catheterization in guiding stent placement. However, the use of coronary angiography in stent implantation in coronary artery disease intervention is limited by several drawbacks, such as the potential of observer bias and interobserver variability. Further technical limitations in angiography exist such as suboptimal visualization of a particular location and angiography only providing information about the contour of the vascular lumen. Angiography does not provide further information regarding the components of the vascular wall being assessed. Angiography is often limited in its ability to detect significant stenosis in the left main coronary artery [1], as there is frequently no reference segment in the left main (LM). It is also limited in ability to analyze the ostial part of the left anterior descending (LAD) and the left circumflex (LCx) because of overlapping anatomy in fluoroscopy, and the planned strategy of percutaneous coronary intervention (PCI) can be significantly altered, for example one or two stents strategy depends on ostial LCx or LAD. Ostial of LM is often not clearly visualized by angiography alone. Because of an unclear reference diameter, accurate sizing of the stent is often challenging. Angiography alone is also limited in its ability to accurately detect stent expansion, which is the most important predictor for in-stent restenosis and in-stent thrombosis [2].

Intravascular ultrasound (IVUS) is an intravascular imaging technique that provides information regarding wall composition on coronary vascular lesions. IVUS allows visualization of the coronary arterial wall using a transducer at the end of the catheter. This transducer emits ultrasound waves which will be reflected by the surroundings and generate detailed information regarding the tunica intima, media, and adventitia of the vessel [3, 4]. Traditionally the usage of coronary angiography only provided information on stenotic vs non-stenotic segments in the coronary arteries. IVUS allows further details to be visualized such as types of plaque, dissections, and calcium depositions [5]. IVUS has also demonstrated that the normal areas on angiography are often markedly abnormal, thus redefining the known extent of atherosclerosis in patients with Coronary Artery Disease (CAD) [5]. Meanwhile, the ability of the angiography to assess the extent of atherosclerotic disease is limited because atherosclerosis has a diffuse nature, intricate shapes within the lumen, and can enlarge coronary vessels [6]. IVUS can provide information for appropriate intervention strategies, for example heavily calcified plaque requiring plaque modification prior to stenting or strategy based on ostial lesions found in side-branches and LM. Additionally, IVUS can accurately detect complications such as geographical miss, stent underexpansion, edge dissection, stent protrusion into the aorta, stent deformation after final kissing balloon dilation, and tissue protrusion into the stent. The advantage of IVUS in cardiovascular outcomes is achieved by the ability to detect abnormalities that were not obvious by angiography. The common angiography procedure has limited ability to detect early atherogenesis in the coronary system. In this meta-analysis we analyzed studies that employed IVUS as a guidance in stent placement in percutaneous coronary interventions. Patients with left main coronary artery disease (LMCAD) tend to have a worse prognosis due to the large ischemic area, thus effective stent placement would benefit this patient group greatly.

It is not surprising that several trials have already proved the superiority of IVUS-guided left main PCI over angiography alone guidance. However, despite these results, the penetration of IVUS guidance of LM PCI is still low. Our meta-analysis aims to evaluate and summarize the current evidence on whether IVUS-guided drug-eluting stent (DES) placement resulted in a better outcome than the angiography-guided DES placement in patients with LMCA disease.

## 2. Methods

This meta-analysis adheres to the Preferred Reporting Items for Systematic Reviews and Meta-analysis (PRISMA) guideline.

### 2.1 Literature Searching 

Literature searching was performed using Scopus, PubMed, Embase, and Clinicaltrials.gov for “(IVUS OR intravascular ultrasound) AND (Angiography) AND (left main coronary artery OR left main disease)” from the inception of the databases until 18 August 2023. Screening for title/abstract was conducted by two independent authors. Any disagreements that arose were resolved by formal discussion.

### 2.2 Inclusion and Exclusion Criteria

The inclusion criteria for this study were: (1) randomized controlled trials (RCTs) and propensity-matched or multivariable-adjusted observational studies reporting IVUS-guided DES placement versus angiography-guided DES placement in patients with LMCA disease and (2) reporting long-term outcomes of cardiovascular mortality or all-cause mortality or target lesion revascularization or myocardial infarction or stent thrombosis.

Exclusion criteria were as follows: (1) editorial or commentaries, (2) review articles, (3) conference papers, (4) letters, and (5) abstracts-only publication. We imposed no language restrictions.

### 2.3 Outcome

The intervention and control group in our study are patients undergoing IVUS-guided DES placement and the patients undergoing angiography alone-guided DES placement, respectively. Outcomes measured were all-cause mortality, cardiovascular mortality, target lesion revascularization, myocardial infarction, and stent thrombosis. Cardiovascular mortality is defined as death due to cardiovascular etiology, while all-cause mortality is death regardless of the cause. Myocardial infarction is defined according to the Universal Definition of Myocardial Infarction Expert Consensus Document which is elevated troponin >99th percentile of the upper reference/normal limit (URL). Target lesion revascularization is defined as the need for revascularization in the patient. Stent thrombosis is defined as thrombotic occlusion of a coronary stent. The hazard ratio (HR) represents the effect estimate. All of the outcomes were long-term, from in-hospital mortality to outpatient follow-up. The period of follow-up varied across studies ranging from several days to 10 years.

### 2.4 Statitical Analysis

Data regarding the baseline characteristics and related outcomes were extracted from the studies by two independent authors. These data include author, study design, inclusion criteria, sample size, comorbidities, age, sex, and the outcome within both the groups. Any disagreements that arose were resolved by formal discussion.

For the non-randomized studies, a risk of bias assessment was conducted by two independent authors using the Newcastle-Ottawa Scale (NOS) [7], while the Cochrane Risk of Bias Assessment was used for RCTs [8]. Any disagreements that arose were resolved by formal discussion.

We performed the meta-analysis using Review Manager software version 5.4.1 (Cochrane Collaboration, Copenhagen, Denmark). A generic inverse variance method was used to pool the log [Hazard Ratio] along with its standard error, the outcome was reported as HR. Heterogeneity between studies was analyzed using the $I^{2}$ statistics. Values above 50% and a *p*-value < 0.10 indicated heterogeneity. The random-effect model was used if the pooled effect estimate was significant for heterogeneity and fixed-effect model was used if there is no significant heterogeneity. For the effect estimates, *p* -values of ≤0.05 were considered significant.

## 3. Results

### 3.1 Baseline Characteristics

**Fig. 1.**
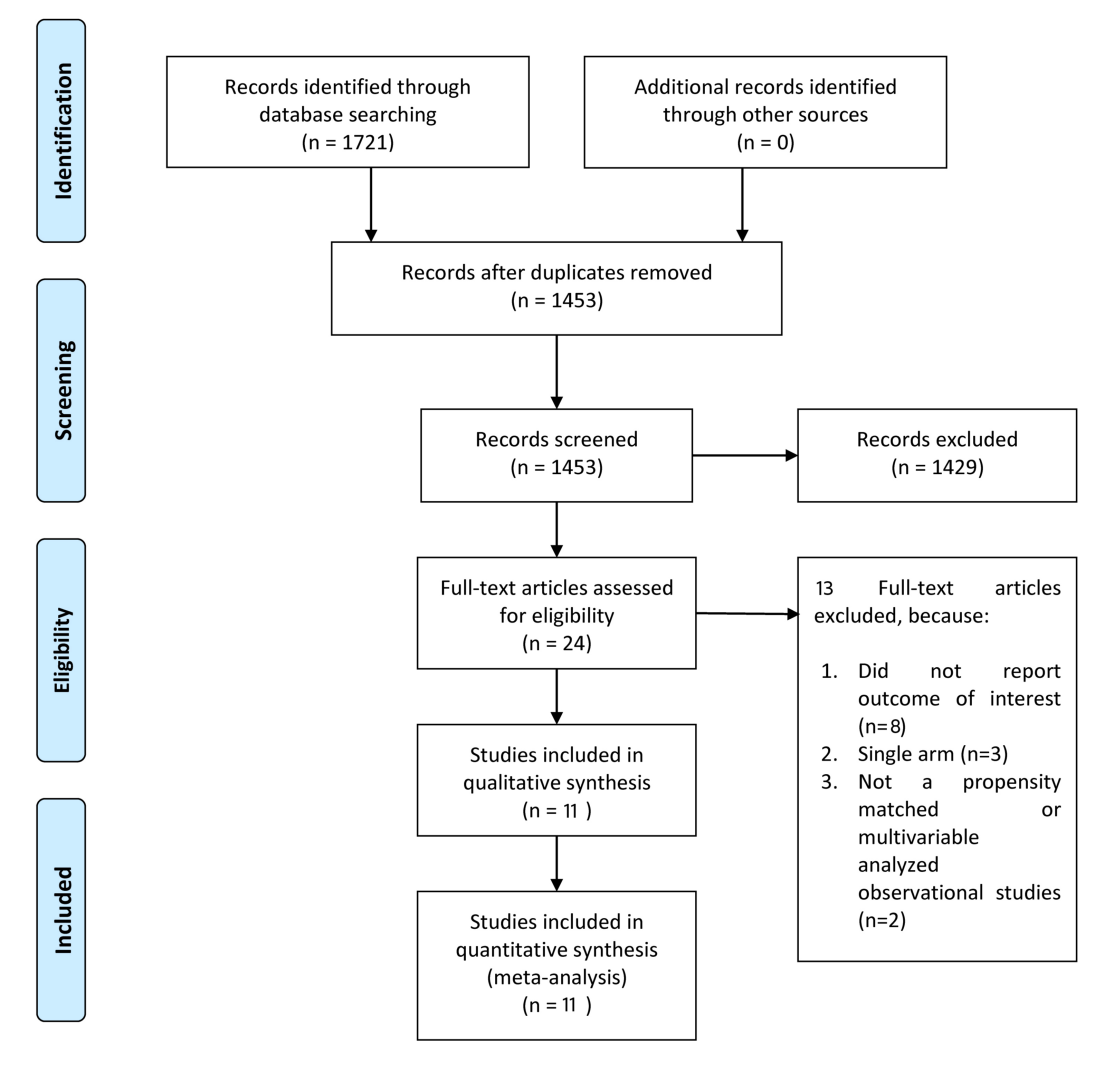
**Preferred Reporting Items for Systematic Reviews and Meta-analysis (PRISMA) Flowchart**.

A total of 11 studies involving 24,103 patients were included in this study (Fig. 1). 2 RCTs and 9 observational studies were included in this analysis. Table 1 (Ref. [9, 10, 11, 12, 13, 14, 15, 16, 17, 18, 19]) summarizes baseline characteristics of the included studies (Table 1 and Fig. 1).

**Table 1.** **Baseline characteristics of the included studies**.

Authors	Study	Sample size	Age (mean, years)	Male (%)	Diabetes (%)	Hypertension (%)	Heart failure (%)	NOS
Andell *et al*. [16] 2017	PS-Matched	680	72	73	25	74	9	9
Choi *et al*. [9] 2019	PS-Matched	6005	63	75	47	60	NA	8
Gao *et al*. [11] 2014	PS-Matched	1016	67	79	33	72	20	8
Hernandez *et al*. [10] 2014	PS-Matched	1010	67	79	35	66	NA	8
Kim *et al*. [12] 2017	PS-Matched	196	64	76	41	66	NA	8
Kinnaird *et al*. [17] 2020	PS-Matched	11,264	70	71	24	64	NA	9
Ladwiniec *et al*. [18] 2020	RCT (Post Hoc)	599	67	81	15	65	NA	8
Liu *et al*. [13] 2019	RCT	336	65	64	32	71	19	RoB*
Park *et al*. [19] 2009	PS-Matched	975	61	70	28	49	1	8
Tan *et al*. [14] 2015	RCT	123	76	66	32	44	NA	RoB*
Tian *et al*. [15] 2017	PS-Matched	1899	60	79	26	56	NA	8

*Unclear Randomization and allocation concealment, single-blind study. PS-Matched, propensity score matched; NA, not available; NOS, newcastle-ottawa scale; RCT, randomized controlled trials; RoB, risk of bias.

### 3.2 Outcomes

**Fig. 2.**
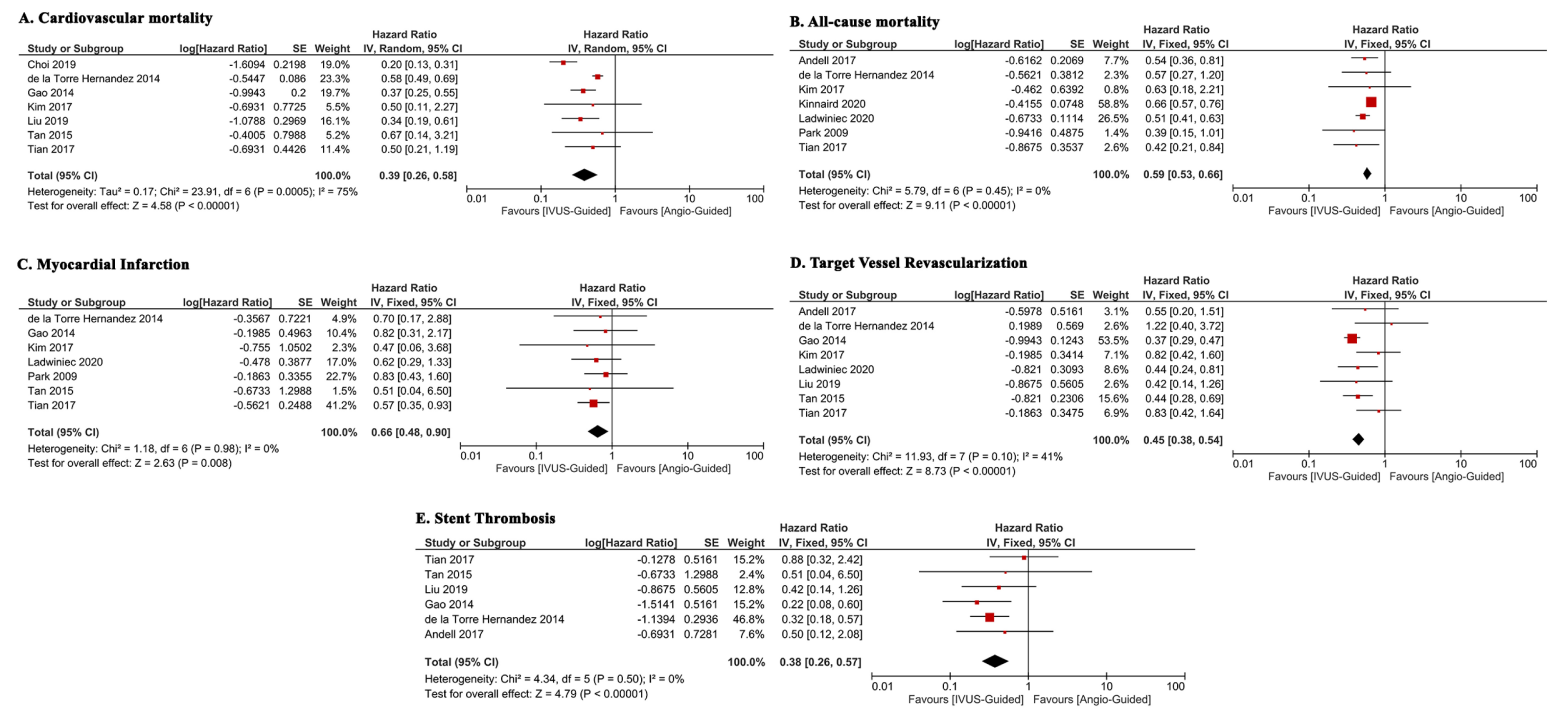
**Meta-analysis of patient outcomes**. (A) Cardiovascular mortality. (B) All-cause mortality. (C) Myocardial Infarction. (D) Target Vessel Revascularization. (E) Stent Thrombosis. SE, standard error; IVUS, intravascular ultrasound.

IVUS-guided DES placement was associated with lower cardiovascular mortality (HR 0.39 [95% CI 0.26, 0.58], *p* < 0.001; $I^{2}$: 75%, *p* < 0.001) [9, 10, 11, 12, 13, 14, 15] (Fig. 2A) and all-cause mortality (HR 0.59 [95% CI 0.53, 0.66], *p* < 0.001; $I^{2}$: 0%, *p* = 0.45) [10, 12, 15, 16, 17, 18, 19] (Fig. 2B) compared to angiography alone guided DES placement. The group receiving IVUS-guided DES placement had a lower incidence of myocardial infarction (HR 0.66 [95% CI 0.48, 0.90], *p* = 0.008; $I^{2}$: 0%, *p* = 0.98) [10, 11, 12, 14, 15, 18, 19] (Fig. 2C) compared to those receiving angiography alone guided DES placement. IVUS-guided DES placement had a significantly lower rate of target lesion revascularization (HR 0.45 [95% CI 0.38, 0.54], *p* < 0.001; $I^{2}$: 41%, *p* = 0.10) [10, 11, 12, 13, 14, 15, 16, 18] (Fig. 2D) and stent thrombosis (HR 0.38 [95% CI 0.26, 0.57], *p* < 0.001; $I^{2}$: 0%, *p* = 0.50) [10, 11, 13, 14, 15, 16] (Fig. 2E).

### 3.3 Publication Bias

**Fig. 3.**
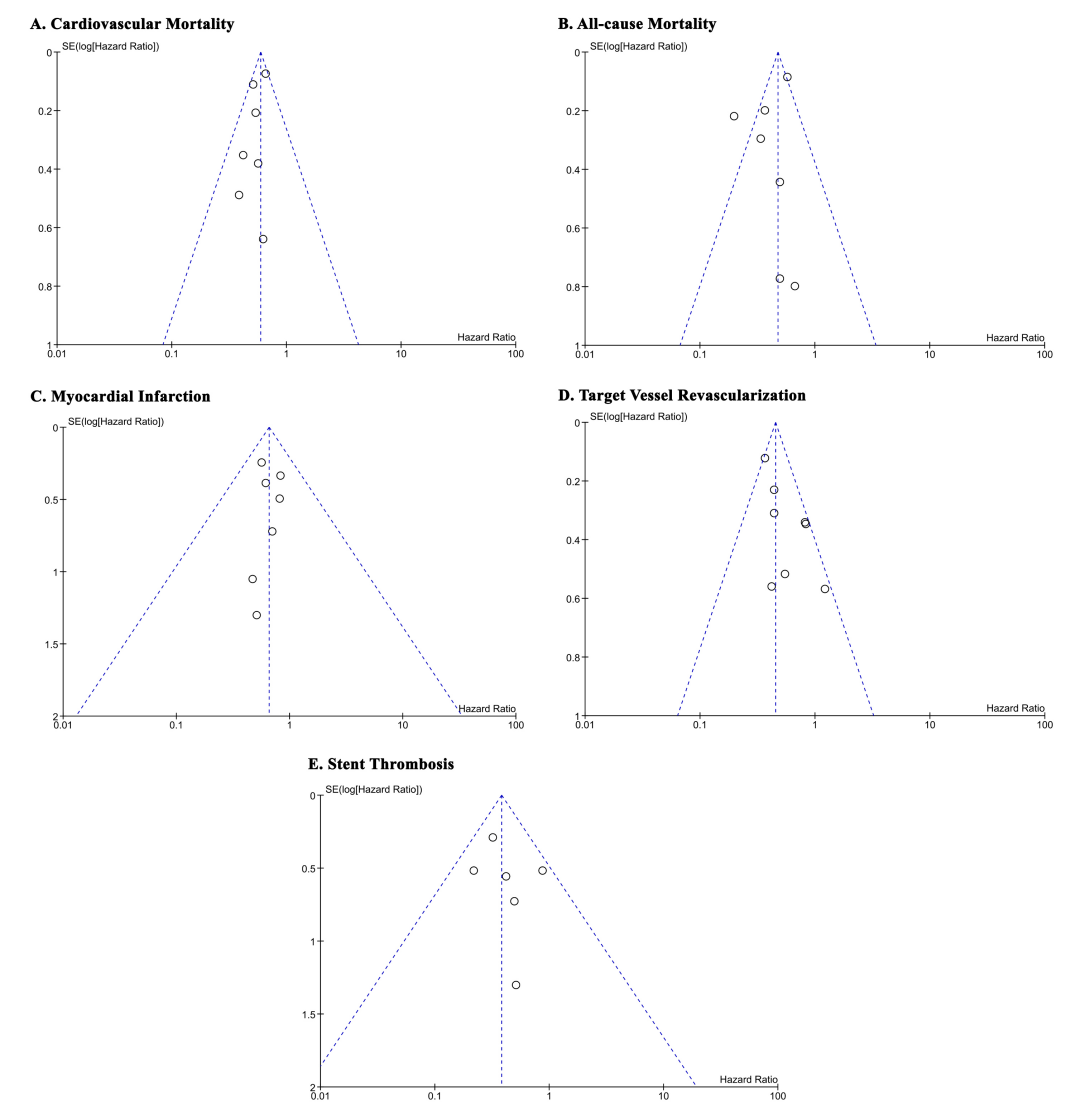
**Funnel-plot analysis**. (A) Cardiovascular mortality. (B) All-cause mortality. (C) Myocardial Infarction. (D) Target Lesion Revascularization. (E) Stent Thrombosis. SE, standard error.

The risk of bias for RCT and observational studies is in Table 1. The Egger’s test was non-significant for small-study effects for cardiovascular mortality (*p* = 0.710), all-cause mortality (*p* = 0.316), myocardial infarction (*p* = 0.993), and stent thrombosis (*p* = 0.495), but not for target vessel revascularization (*p* = 0.010). Funnel-plot analysis was displayed in Fig. 3.

## 4. Discussion

This meta-analysis showed that IVUS-guided DES placement had lower cardiovascular mortality, all-cause mortality, target lesion revascularization, myocardial infarction, and stent thrombosis than angiography alone guided DES placement in both stable and unstable coronary artery disease.

The advantage of IVUS in cardiovascular outcomes is achieved by the ability to detect abnormalities that were not identified or occultly identified by angiography. The common angiography procedure has limited ability to detect early atherogenesis in the coronary system. Coronary angiography also does not offer means to quantify plaque burden or compensatory arterial remodeling [20]. Detection of angiographically occult CAD has significant prognostic value. The results of this meta-analysis are similar to a previous PROSPECT study (Providing Regional Observations to Study Predictors of Events in Coronary Tree), that found major adverse cardiac events after a median follow-up of 3.4 years in patients with lesions deemed non-culprit by initial angiography. These lesions were responsible for future cardiovascular events showed mild abnormalities on angiography at baseline [21].

IVUS offers details regarding the size and structure of the affected vessel, composition and morphology of plaque, and extent of disease. These IVUS-derived parameters are relevant in coronary stenting which will include measurement of stent size, identification of stent morphological change including enlargement and apposition, and avoidance of misplacement at the target location [22].

The capability of IVUS in detecting occult angiographical disease was also observed in patients with stable coronary disease. While the value of PCI in stable coronary disease is highly debated, using IVUS-guided PCI, the Japan Stable Angina Pectoris (JSAP) trial showed a reduction in the incidence of Acute Coronary Syndrome (ACS) in IVUS-guided PCI patients compared to patients receiving medical therapy [23]. On the other hand, this benefit was not seen in the Clinical Outcomes Utilizing Revascularization and Aggressive Drug Evaluation (COURAGE) trial in which only conventional angiographically guided PCI was performed [24].

The result of this meta-analysis is similar to the results of the ADAPT-DES study (The Assessment of Dual Antiplatelet Therapy With Drug-Eluting Stents) which observed a significantly lower rate of myocardial infarction, stent thrombosis, and major adverse cardiovascular event (MACE) in patients with IVUS guided procedures versus angiography alone at 1-year post-PCI follow up, this benefit was particularly seen in ACS patients [25].

In this study, we observed the protective effects of IVUS against future myocardial infarction (HR 0.66 [95% CI 0.48, 0.90], *p* = 0.008; $I^{2}$: 0%, *p* = 0.98) and stent thrombosis (HR 0.38 [95% CI 0.26, 0.57], *p* < 0.001; $I^{2}$: 0%, *p* = 0.50). This effect seems to be mediated by the ability of IVUS to detect stents under expansion at the time of placement as previously described by a study by Kang *et al*. [26], in which the author found that patients with under expansion of >1 segment had a significantly lower survival rate (2-year MACE free) than patients with no under expansion (90 ± 3% and 98 ± 1%, respectively; log-rank *p* < 0.001). Furthermore, IVUS assessed LMCA DES under expansion were a good predictor of MACE using regression analysis (adjusted HR = 5.56; 95% CI, 1.99–15.49; *p* = 0.001).

Based on the results of this meta-analysis and comparable studies, IVUS shows potential in guiding PCI procedures, patients with IVUS-guided PCI shows lower rates of mortality, stent thrombosis, and myocardial infarction, with these benefits IVUS shows potential in detecting subtle plaques in the left main section of the coronary artery. The expected increase in the cost of procedure/care with the usage of IVUS in LM lesion patients are justified by the potential benefit of using this guidance. PCI guidance using IVUS should be performed by adequately trained professionals to obtain optimal results.

The limitation of this study was the different study designs and follow-up duration of the included studies. 2 RCT and 9 observational studies were analysed for meta-analysis. Studies which was largely different to the rest may responsible for the bias in this meta analysis.

## 5. Conclusions

This meta-analysis showed that PCI using IVUS guidance resulted in lower all-cause mortality, cardiovascular mortality, target lesion revascularization, myocardial infarction, and stent thrombosis compared to angiography guidance alone.

## Data Availability

The datasets in this meta-analysis are available upon reasonable request.

## Supplementary Material

Supplementary material associated with this article can be found, in the online version, at https://doi.org/10.31083/j.rcm2501032.
